# Different Techniques for Facilitating Percutaneous Dilatational Tracheostomy in Critically Ill Patients and Their Impact on Outcomes: A Single-Center Retrospective Cohort Study

**DOI:** 10.3390/jcm15114096

**Published:** 2026-05-26

**Authors:** Elif Selva Taş, Mehmet Salih Sevdi, Rasim Onur Karaoğlu, Ali Özalp, Süha Bozbay, İsmail Kayaalp, Serdar Demirgan, Ayşin Selcan

**Affiliations:** 1 Department of Anesthesiology and Reanimation, Bağcılar Training and Research Hospital, Istanbul 34200, Türkiye; elifselvabayram@gmail.com (E.S.T.); salihsevdi@yahoo.com (M.S.S.); aliozalp1164@gmail.com (A.Ö.); serdar.demirgan@hotmail.com (S.D.); aysinselcan@gmail.com (A.S.); 2 Department of Intensive Care Medicine, Bağcılar Training and Research Hospital, Istanbul 34200, Türkiye; drshbzby@gmail.com (S.B.);

**Keywords:** tracheostomy, percutaneous dilational tracheostomy, ultrasound, bronchoscopy, mortality

## Abstract

**Objective:** Prolonged intubation in critically ill patients can lead to airway injury and infection. Early percutaneous dilational tracheostomy (PDT) is preferred in the intensive care unit (ICU), but the optimal guidance technique remains debated. We aimed to compare outcomes of PDT facilitated by three different guidance techniques: blind landmark, bronchoscopy-guided, and ultrasound-guided. **Methods**: This single-center retrospective cohort study included adults who underwent bedside PDT between 2013 and 2023. Patients were grouped by technique: bronchoscopy-guided (Group A), ultrasound-guided (Group B), and blind landmark technique (Group C). Demographics, illness severity, procedural details, complications, infection rates, and outcomes including mortality were analyzed. **Results**: A total of 254 patients were analyzed (118 bronchoscopy-guided, 50 ultrasound-guided, and 86 blind). Baseline illness severity scores were comparable among groups, although admission diagnosis distributions differed significantly. Tracheostomy-related complications occurred in 32% of patients, most commonly minor bleeding (~23%), with no significant intergroup differences. Early mortality following PDT (≤72 h) and post-procedural complication rates were similar among techniques. Pneumothorax and tracheoesophageal fistula were rare events. Ventilator liberation and decannulation rates did not differ significantly between groups. ICU and hospital mortality were numerically higher in the blind group; however, these findings should be interpreted cautiously because of the retrospective design and differences in admission diagnoses and case-mix between groups. **Conclusions**: All three PDT guidance techniques showed comparable procedural safety, with no significant differences in early complications, infection rates, or ventilator liberation. Although blind PDT was associated with higher observed mortality, this cannot be causally attributed to the technique, given the retrospective design and potential confounders. Ultrasound guidance offers practical procedural advantages and should be considered where available, pending prospective randomized confirmation.

## 1. Introduction

Tracheostomy is commonly used in critically ill patients who require prolonged mechanical ventilation to secure the airway, reduce the risks of extended translaryngeal intubation, and facilitate weaning [1]. Prolonged endotracheal intubation, particularly beyond 10–14 days, is associated with a higher risk of complications such as ventilator-associated pneumonia (VAP), aspiration, laryngeal injury, vocal cord dysfunction, and tracheal stenosis [1,2]. Therefore, clinical guidelines recommend considering tracheostomy within the first 1–2 weeks of ventilation in appropriate patients to improve safety and outcomes [3].

Over the past few decades, percutaneous dilatational tracheostomy (PDT) has replaced surgical tracheostomy as the preferred technique in many intensive care units [4,5]. PDT can be performed at the bedside using a guidewire and dilator system without the need for surgical dissection, thereby avoiding operating room transfer and reducing cost [4,6]. Large reviews and meta-analyses have confirmed that PDT is at least as safe as surgical tracheostomy, while being associated with lower wound infection and scarring rates [5,6].

The original PDT technique was performed using anatomical landmarks and palpation without direct visualization, also referred to as the “blind” technique [7]. To increase procedural safety, two main adjuncts have been introduced: fiberoptic bronchoscopy and ultrasonography. Fiberoptic bronchoscopy provides real-time intratracheal visualization, confirming correct puncture and guidewire position, and potentially reducing the risk of posterior wall injury or false passage [8,9]. However, bronchoscopy may partially obstruct ventilation, cause hypercapnia, and is not always available in all ICUs, limiting its universal use [10,11].

Ultrasound guidance has more recently been adopted as a valuable alternative. Preprocedural ultrasonography of the neck can identify the tracheal midline, tracheal rings, and adjacent vascular structures, thereby helping to select the safest puncture site [12,13]. Real-time ultrasound guidance allows direct visualization of the needle and dilator during insertion, which has been shown to improve first-pass success and reduce minor bleeding or misplacement [14,15]. Importantly, ultrasound does not interfere with ventilation and is readily available at the bedside in most modern ICUs [16]. Some studies have even suggested that a combined approach using both ultrasound and bronchoscopy may maximize safety by integrating vascular mapping with intratracheal confirmation [17].

In practice, the choice of guidance varies across institutions and clinicians. While many centers routinely perform PDT with bronchoscopic assistance, others increasingly use ultrasound or, in selected straightforward cases, continue to apply the landmark technique [18,19]. Despite multiple investigations, data remain limited regarding whether these different techniques influence not only procedural complications but also broader patient-centered outcomes such as infection, ventilator days, ICU stay, and survival [20].

The present study evaluates outcomes of PDT facilitated by three different guidance techniques (bronchoscopy-guided, ultrasound-guided, and blind landmark) in a large adult ICU cohort, focusing on complication rates, infection, length of stay, and mortality.

## 2. Materials and Methods

Study Design and Setting: This study was a retrospective, observational cohort analysis conducted at a tertiary-care university hospital ICU. The ICU is a multidisciplinary adult intensive care unit with a high volume of mechanically ventilated patients, including medical, surgical, and trauma cases. This study was approved by the Bağcılar Training and Research Hospital Non-Interventional Clinical Research Ethics Committee (Decision No: 2024/02/06/020, Date: 22 February 2024). Due to the retrospective design, the requirement for informed consent was waived by the ethics committee. The study adhered to the STROBE guidelines for observational studies. No formal sample size calculation was performed because this was a retrospective cohort including all eligible patients within the 10-year period. Thus, the study size was determined by available cases rather than a priori power analysis. Multivariable logistic regression was performed to identify independent predictors of hospital mortality. Variables included in the model were age, sex, APACHE II score, primary admission diagnosis category (medical, surgical, trauma), SOFA score at the time of tracheostomy, tracheostomy timing (days from intubation to procedure), and year of procedure (to account for temporal changes in practice). Guidance technique (bronchoscopy, ultrasound, or blind) was entered as the main exposure variable.

A post hoc power analysis based on the observed difference in hospital mortality between the blind and ultrasound groups (87% vs. 68%) indicated that approximately 40–50 patients per group would provide 80% power to detect such a difference at a two-sided alpha of 0.05. Since our sample included 86 blind cases and 118 bronchoscopy cases, the study was adequately powered for the primary outcome of mortality.

**Figure 1:**
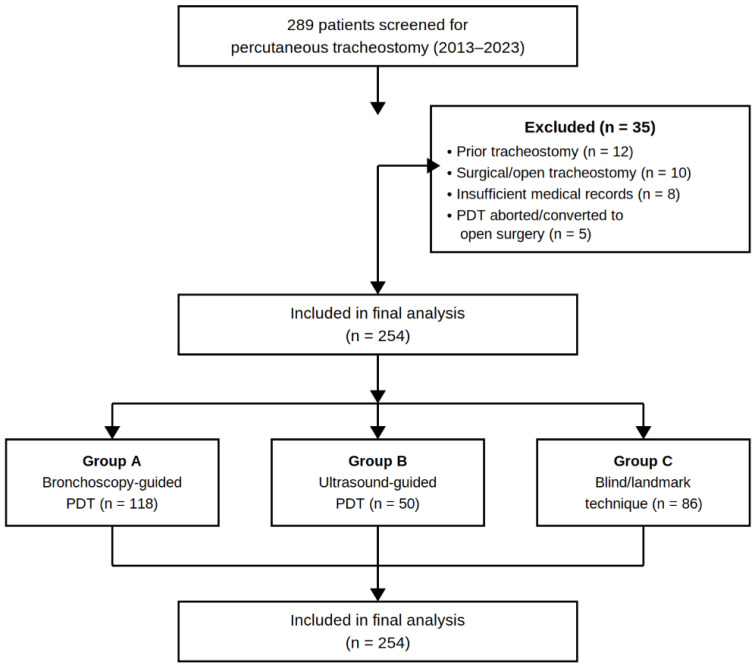
Study flow diagram.

Patient Selection: We screened all patients who underwent a percutaneous dilational tracheostomy (PDT) in our ICU between 1 December 2013, and 31 December 2023. Inclusion criteria were: age ≥ 18 years; intubated and ventilated in ICU at the time of tracheostomy; tracheostomy performed percutaneously at the bedside by the ICU team (using a standard single-dilator Ciaglia technique). Exclusion criteria were: patients who had a prior tracheostomy before this ICU admission; patients who underwent surgical tracheostomy (open technique) instead of percutaneous; patients for whom adequate medical records or procedural details were not available; and patients in whom the PDT was aborted or converted to surgical intra-operatively. A total of 289 patients were screened, of whom 35 were excluded: 12 had a prior tracheostomy, 10 underwent surgical/open tracheostomy, 8 had insufficient medical records, and 5 had a PDT that was aborted or converted to open surgical tracheostomy intra-operatively. The remaining 254 cases were included in the final analysis (Figure 1). We did not exclude patients due to partial missing values; only cases with completely insufficient records were excluded a priori, so no data imputation was required. Missing data were therefore not a source of bias, as incomplete records were excluded before analysis.

PDT Guidance Technique Groups: Patients were categorized into three groups according to the technique used for PDT:

- Group A—Bronchoscopy-guided PDT: Fiberoptic bronchoscopy was used throughout the procedure. An adult flexible bronchoscope was inserted via the endotracheal tube to visualize the anterior tracheal wall. The bronchoscope allowed real-time confirmation of needle entry and guidewire position in the trachea. The Ciaglia single-dilator method was then completed under bronchoscopic visualization. This group represents the FOB-guided standard.
- Group B—Ultrasound-guided PDT: Real-time ultrasonography of the neck was utilized during the procedure. A linear ultrasound probe was used to identify tracheal rings, midline, and vessels. The puncture site was chosen and confirmed with ultrasound, and the needle was inserted with ultrasound guidance (either in-plane longitudinal or out-of-plane transverse view). No bronchoscope was used in this group. This represents the US-guided technique.
- Group C—Blind PDT: The percutaneous tracheostomy was done using anatomical landmarks and palpation only, with no bronchoscopic or ultrasound guidance. The operator identified the cricoid and tracheal rings by landmark, confirmed endotracheal tube withdrawal to near the cords (by counting pilot balloon markings), and performed the needle puncture blindly. This reflects the traditional landmark technique.

The decision of which technique to use was not randomized; it was determined by operator preference, availability of equipment, and patient factors. In our ICU, bronchoscopy-guided PDT was generally standard during daytime hours when a bronchoscopist was available, whereas ultrasound guidance became increasingly available after ~2018 with trained intensivists. The blind technique was often utilized in earlier years or off-hours. This pragmatic non-random allocation is acknowledged and addressed in the analysis.

Procedure Protocol: All tracheostomies were performed at bedside under aseptic conditions. Patients were adequately sedated and/or paralyzed for the procedure as per standard ICU practice. The Ciaglia Blue Rhino single-dilator kit (Cook Critical Care) or an equivalent percutaneous tracheostomy kit was used for all cases. After skin prep and local anesthesia infiltration, the trachea was punctured (typically between the 2nd and 3rd tracheal rings) with a needle and introducer cannula. A guidewire was inserted into the trachea, and sequential dilation (single-step dilation in most cases) was performed to create the tract. A tracheostomy tube (usually size 8.0 cuffed) was inserted over the dilator and guidewire into the trachea. Correct placement was confirmed by capnography and bilateral breath sounds in all cases, and by bronchoscope in Group A or ultrasound visualization of the tube in the trachea in some Group B cases. Immediate complications (e.g., significant bleeding, pneumothorax) were assessed and managed at the bedside. All patients received a post-procedure chest X-ray to check for pneumothorax or other complications.

Data Collection: We extracted data from electronic medical records, procedure notes, and ICU flow sheets. Collected variables included: demographics (age, sex), underlying conditions (presence of any major chronic comorbidities such as COPD, heart failure, etc.), and severity of illness scores at ICU admission (Acute Physiology and Chronic Health Evaluation II—APACHE II; and Sequential Organ Failure Assessment—SOFA). We recorded the primary indication for ICU admission for each patient (e.g., post-operative respiratory failure, sepsis/septic shock, neurologic illness, trauma, etc.). We also noted coagulation status at the time of tracheostomy (platelet count, INR) and whether any blood products were given prophylactically (e.g., platelet transfusion if thrombocytopenia).

For the tracheostomy procedure itself, we documented the technique used (A, B, or C as defined), the day of ICU stay on which tracheostomy was performed, and any immediate technical difficulties or aborted attempts. We recorded all procedure-related complications, subdivided into intra-procedural and post-procedural. Complications were defined and categorized as follows:

- Bleeding: any hemorrhage from the tracheostomy site. We classified bleeding as minor (self-limited or requiring only local pressure) versus major bleeding (requiring surgical exploration or transfusion). We also recorded if any patient developed a delayed hemorrhage due to arterial fistula (a rare but life-threatening complication).
- Accidental paratracheal placement: any evidence that the tracheostomy tube was not in the trachea (if it happened, it would be immediate and corrected—none persisted in our data).
- Pneumothorax: new air in the pleural space on post-procedure imaging.
- Subcutaneous emphysema: significant subcutaneous air in the neck or chest wall.
- Tracheoesophageal fistula: diagnosed if there was clinical evidence of a persistent communication (air leak via mouth, aspiration via trach, etc.) later confirmed by endoscopy/radiology.
- Posterior tracheal wall injury: not explicitly documented, but any mention of difficulty passing a dilator or bronchoscope, or seeing injury.
- Cardiac arrest: if the patient had a cardiac arrest during or within 30 min after the procedure.

We also noted if multiple attempts (multiple needle sticks) were required or if the procedure was converted to an open tracheostomy (none were in this series).

We then collected outcome data after tracheostomy. This included: the development of any new respiratory infections (such as ventilator-associated pneumonia or tracheobronchitis) post-tracheostomy, defined by positive respiratory cultures and clinical signs requiring antibiotic treatment. We recorded whether patients required vasopressors (inotropes) for hemodynamic support after tracheostomy (beyond what they needed before) and whether they required any blood transfusions specifically due to the procedure (e.g., to treat bleeding). We also documented the total duration of mechanical ventilation for each patient (in days), subdivided into days before tracheostomy and days after tracheostomy until ventilator weaning. The ICU length of stay and hospital length of stay were recorded (for survivors, until discharge; for non-survivors, until death). We noted the outcome at ICU discharge (still intubated on ventilator, weaned to spontaneous breathing with trach collar, decannulated, or deceased) and at hospital discharge (alive and discharged, or died in hospital). For those discharged, we tracked if the patient was discharged with tracheostomy in place (to a chronic care facility or home on ventilator) or if they were decannulated prior to discharge. Finally, we examined the mortality data: ICU mortality and in-hospital mortality. For patients who died, we reviewed their primary cause of death from medical records (categorized as sepsis, multi-organ failure, refractory respiratory failure, major hemorrhage, or neurologic death).

### Statistical Analysis

Data were analyzed using SPSS version 27. Descriptive statistics were calculated for all variables. Continuous variables were presented as mean ± standard deviation (SD) if normally distributed, or as median [min–max] if not. Categorical variables were expressed as counts and percentages. Normality was assessed with the Kolmogorov–Smirnov test.

For comparisons among the three groups, one-way ANOVA (with Tukey post hoc test) was used for continuous variables with approximately normal distribution, and the Kruskal–Wallis test (with Mann–Whitney U for pairwise comparisons) was used for non-parametric data. For categorical variables, chi-square or Fisher’s exact tests were performed as appropriate. When overall group differences were significant, Bonferroni-adjusted pairwise proportion comparisons were conducted to determine which groups differed.

In addition to *p*-values, effect sizes for key binary outcomes (such as mortality and complications) were expressed as risk ratios (RR) or odds ratios (OR) with corresponding 95% confidence intervals (CIs). For outcomes with zero events, the Haldane–Anscombe correction (0.5) was applied. A two-tailed *p* < 0.05 was considered statistically significant. Adjusted significance thresholds were applied for multiple comparisons where appropriate.

## 3. Results

### 3.1. Patient Characteristics

A total of 289 patients were screened for percutaneous tracheostomy between 2013 and 2023. After exclusion of 35 patients (12 with prior tracheostomy, 10 who underwent surgical tracheostomy, 8 with insufficient records, and 5 in whom PDT was aborted or converted to open surgery intra-operatively), 254 patients were included in the final analysis. The cohort had a mean age of 64.5 years (±16.6), and 62.2% were male. The mean APACHE II score at ICU admission was 22.1 (±7.4), and the median SOFA score was 5. ICU admission diagnoses were: 39% post-operative respiratory failure, 27% sepsis/septic shock, 11% acute neurological events, 8% acute cardiac failure, and 15% miscellaneous causes. All patients were intubated and ventilated at the time of tracheostomy.

Baseline characteristics were similar across groups (Group A: FOB-guided, n = 118; Group B: US-guided, n = 50; Group C: blind, n = 86) as shown in Table 1. Age (63.2, 63.0, 67.1 years; *p* = 0.20), sex distribution (~60–65% male, *p* = 0.62), and severity scores (APACHE II: 21, 23, 23; *p* = 0.14; SOFA: 5, 6, 6; *p* = 0.47) did not differ significantly. Admission diagnosis distribution varied significantly among groups (*p* < 0.001).

### 3.2. Timing of Tracheostomy

The median time from ICU admission to tracheostomy was 16 days (IQR 10–22). Group B (ultrasound) had later tracheostomies (median 18 days) compared to Group C (median 14 days, *p* = 0.008). Group A was intermediate (median 15 days).

### 3.3. Procedural Complications

Overall, 81 patients (31.9%) experienced at least one complication. The most common was bleeding, occurring in 58 patients (22.8%). Five patients (1.97%) required transfusion, and two patients (0.8%) developed fatal late tracheo-innominate fistulas. Immediate peri-procedural bleeding rates were similar across groups (Group A: 22.9%; Group B: 22.0%; Group C: 23.3%; *p* = 0.99; RR 1.06, 95% CI 0.55–2.02) (Table 2).

Other complications were infrequent. Pneumothorax occurred in 3.5% vs. 0% (*p* = 0.156; RR 4.10, 95% CI 0.22–77.85, adjusted with 0.5 correction for zero cell). Subcutaneous emphysema was observed in three patients (1.2%): two in Group C and one in Group A (*p* = 0.25; RR 2.93, 95% CI 0.14–59.86, 0.5 correction). Tracheoesophageal fistula occurred in seven patients (2.8%): three in Group B, three in Group C, and one in Group A (*p* = 0.29; RR 0.58, 95% CI 0.12–2.77). Cardiac arrest occurred in six patients (2.4%): three in Group A, two in Group C, and one in Group B (*p* = 0.097; RR 1.16, 95% CI 0.11–12.50). No tracheal ring fractures, posterior wall perforations, or guidewire misplacements were documented. Overall complication rate was 28.8% in Group A, 30.0% in Group B, and 37.2% in Group C (*p* = 0.424; RR 1.24, 95% CI 0.75–2.05 for blind vs. ultrasound).

### 3.4. Infections and Ventilator Outcomes

Ventilator-associated pneumonia or tracheal infection occurred in 73 patients (28.7%). Incidence was 29% in Group A, 38% in Group B, and 23% in Group C (*p* = 0.187). Successful ventilator liberation was achieved in 155 patients (61%), without significant group differences (*p* = 0.88; RR 0.91, 95% CI 0.69–1.20). Decannulation before hospital discharge was rare (9% overall; *p* = 0.93; RR 0.87, 95% CI 0.15–5.04) (Table 3).

### 3.5. Length of Stay

Median ICU length of stay was 53.5 days in Group A, 49.5 days in Group B, and 32.5 days in Group C (*p* = 0.017). Median hospital stay was 66 days in Group A, 77 days in Group B and 58.5 days in Group C (*p* = 0.046) (Table 3).

### 3.6. Mortality

ICU mortality was 53.5% overall (Table 3). ICU and hospital mortality rates numerically differed among groups, with higher observed mortality in the blind technique group compared with the bronchoscopy- and ultrasound-guided groups. However, early mortality following PDT (≤72 h) was similar between techniques. Discharge survival was higher in the ultrasound-guided group (32.0% vs. 12.8% in the blind group; RR 0.40, 95% CI 0.20–0.79; *p* = 0.006). In multivariable logistic regression analysis adjusted for age, APACHE II score, SOFA score, admission diagnosis category, tracheostomy timing, and year of procedure, the higher observed in-hospital mortality in the blind technique group remained numerically greater; however, this finding should be interpreted cautiously because residual confounding, selection bias, and differences in case-mix may still have substantially influenced outcomes. Among patients who died in the ICU, 12 (8.8%) were diagnosed with brain death, following which life-sustaining treatment was withdrawn in accordance with institutional protocol and family consent. The remaining deaths were attributable to disease progression.

## 4. Discussion

In this retrospective study of 254 ICU patients undergoing percutaneous tracheostomy (PDT), we compared outcomes of PDT facilitated by bronchoscopy-guided, ultrasound-guided, and landmark-guided (blind) guidance techniques. All three guidance approaches were technically feasible and demonstrated comparable early procedural safety, early mortality, and complication profiles. Although ICU and hospital mortality differed numerically among groups, these differences are likely influenced by differences in admission diagnoses, case-mix, and temporal practice patterns rather than the PDT guidance technique itself. Our findings, therefore, suggest that the three approaches appear similarly safe in terms of immediate post-procedural outcomes.

The longer median hospital stay observed in the ultrasound group (77 days) should be interpreted in the context of survival rather than as an adverse outcome. Patients in the blind technique group had significantly higher mortality, resulting in shorter apparent hospital stays due to earlier death. The extended hospitalization in the ultrasound group likely reflects greater survival to discharge rather than increased morbidity associated with the technique. Additionally, the ultrasound group underwent tracheostomy later (median 18 vs. 14 days), which independently contributed to longer overall hospitalization.

Our complication rates were broadly similar across groups, consistent with previous trials and meta-analyses reporting low rates of major complications with PDT [12,13,14,15,16,18]. In particular, ultrasound and bronchoscopy had comparable rates of pneumothorax, bleeding, and subcutaneous emphysema, supporting the notion that both are safe alternatives [12,13]. Large reviews have shown that bleeding requiring transfusion occurs in ~2–5% and pneumothorax in <2% of PDTs [14,15,18]. Our findings align with these benchmarks. Ultrasound guidance prevented pneumothorax in our cohort, consistent with meta-analyses reporting improved precision and fewer needle passes with ultrasound [14,16,19].

Despite similar complication profiles, the mortality difference between groups was striking. Blind PDT patients demonstrated numerically higher mortality compared with ultrasound-guided cases; however, this finding should be interpreted cautiously because residual confounding and differences in case-mix may have substantially influenced outcomes despite multivariable adjustment. This may reflect unmeasured confounding, since blind procedures were often performed during emergencies or off-hours. Although data on the number of needle attempts per procedure were not systematically recorded in our retrospective cohort, the literature consistently reports that blind landmark-guided PDT is associated with a higher rate of multiple puncture attempts compared with guided techniques, which may contribute to increased procedural trauma and complications. By enabling real-time visualization, ultrasound likely minimized these risks, which may have contributed to differences in observed outcomes. Prior reviews also highlight ultrasound’s role in improving first-pass success and reducing vascular injury [15,16,19].

Bronchoscopy-guided PDT showed intermediate results. It reliably ensured intratracheal placement and avoided false passages, consistent with earlier studies [7,8,9,10]. However, bronchoscopy may worsen ventilation, increase CO_2_ retention, and raise intracranial pressure in certain patients [8,9]. These physiological considerations may partly explain the observed differences; however, it should be noted that bronchoscopy-guided PDT was more frequently selected for patients with sepsis and neurological diagnoses in our cohort, introducing potential confounding that limits causal interpretation. The observed outcome differences between groups likely reflect differences in patient case-mix rather than a direct effect of the guidance technique itself. Our findings support recent literature suggesting ultrasound can safely replace bronchoscopy in many settings [12,13,20], though combining both may maximize safety in high-risk cases [17].

Our study has limitations. This was a retrospective, non-randomized single-center study, which may limit generalizability. The ultrasound group was smaller (n = 50) and derived from later years, introducing possible time-trend bias. Selection bias due to non-random allocation and evolving clinical practices could also influence results. Nevertheless, patient demographics and illness severity were comparable among groups, suggesting that our findings are broadly representative of high-acuity ICU populations where percutaneous tracheostomy is performed. Some rare complications, such as tracheoesophageal fistula, were too infrequent for meaningful comparison. Mortality differences cannot be assumed causal but highlight that guidance choice may matter in critically ill patients. Another limitation is the absence of a formal a priori sample size calculation; our analysis included all available cases, although post hoc power analysis suggests adequate power for the primary mortality outcome. The high ICU mortality rate (54%) observed in our cohort is consistent with the severity of underlying illness in this population. Formal withdrawal of life-sustaining treatment occurred in 12 patients (8.8% of ICU deaths) following brain death certification. The low decannulation rate (9%) similarly reflects the burden of irreversible comorbidities rather than procedural failure.

Nevertheless, this study adds real-world data from a large cohort including all three techniques. It emphasizes that while PDT is generally safe regardless of method, using guidance, especially ultrasound, may improve procedural safety and first-pass accuracy in high-risk ICU patients.

## 5. Conclusions

In this single-center retrospective comparison of 254 percutaneous tracheostomies over a 10-year period, all three guidance techniques used to facilitate PDT (bronchoscopy-guided, ultrasound-guided, and blind landmark) were technically feasible with comparable procedural complication rates, infection rates, and rates of ventilator liberation. These findings confirm that PDT is a safe bedside procedure regardless of the guidance modality used.

Although ICU and hospital mortality differed numerically among groups, these differences are likely influenced by differences in admission diagnoses, illness severity, case-mix, operator experience, and temporal practice patterns rather than the PDT guidance technique itself. Therefore, given the retrospective and non-randomized design of this study, these findings cannot be interpreted as evidence of a causal relationship between guidance modality and mortality. Early procedural complications, the outcomes most directly linked to technique, were similar across all groups, further supporting this interpretation.

Guided techniques, particularly ultrasound, may offer practical procedural advantages such as real-time vascular mapping and improved first-pass accuracy. Prospective randomized studies are needed to determine whether guidance modality independently influences clinically relevant patient outcomes.

## Figures and Tables

**Table 1:** Baseline characteristics, admission diagnoses, and comorbidities by PDT guidance technique group.

	Group A FOB (n = 118)	Group B US (n = 50)	Group C Blind (n = 86)	Total (n = 254)	*p* Value
DEMOGRAPHICS & SEVERITY					
Age, years (mean ± SD)	63.2 ± 16.6	63.0 ± 15.9	67.1 ± 16.6	64.5 ± 16.6	0.197
Male sex, n (%)	41 (35%)	21 (42%)	34 (40%)	96 (38%)	0.621
APACHE II, median (min–max)	21 (11–41)	23 (10–38)	23 (8–41)	—	0.141
SOFA, median (min–max)	5 (1–17)	6 (1–15)	6 (1–12)	—	0.466
Days to tracheostomy (mean ± SD)	16.5 ± 8.3	19.1 ± 9.9	14.6 ± 6.1	16.4 ± 8.1	0.008 *
ADMISSION DIAGNOSIS, N (%)					
Post-operative	39 (33%)	23 (46%)	36 (42%)	98 (39%)	<0.001
Sepsis/septic shock	36 (31%)	11 (22%)	22 (26%)	69 (27%)	<0.001 *
Neurological	17 (14%)	5 (10%)	7 (8%)	29 (11%)	<0.001
Cardiac	6 (5%)	5 (10%)	9 (10%)	20 (8%)	0.005
Trauma	10 (8%)	0 (0%)	8 (9%)	18 (7%)	<0.001 *
Renal failure	3 (3%)	2 (4%)	3 (3%)	8 (3%)	0.092
Respiratory	3 (3%)	1 (2%)	1 (1%)	5 (2%)	0.102
Other	5 (4%)	3 (6%)	7 (8%)	15 (6%)	—
COMORBIDITIES, N (%)					
Any comorbidity	105 (89%)	46 (92%)	73 (85%)	224 (88%)	0.434
Neurological disease	27 (23%)	11 (22%)	16 (19%)	54 (21%)	<0.001
Cardiac disease	23 (19%)	11 (22%)	17 (20%)	51 (20%)	<0.001
Malignancy	18 (15%)	13 (26%)	15 (17%)	46 (18%)	<0.001
Respiratory disease	14 (12%)	3 (6%)	9 (10%)	26 (10%)	<0.001 *
Renal insufficiency	9 (8%)	3 (6%)	7 (8%)	19 (7%)	0.001
Endocrine disease	9 (8%)	2 (4%)	7 (8%)	18 (7%)	0.001
Hematological disease	3 (3%)	2 (4%)	1 (1%)	6 (2%)	0.097

Values are mean ± SD, median (min–max), or n (%). ANOVA or chi-square unless otherwise noted. * Bonferroni-adjusted *p* < 0.05 for significant pairwise differences. FOB = fiberoptic bronchoscopy; US = ultrasound.

**Table 2:** Percutaneous Tracheostomy Complications by Technique.

Complication	Group A: Bronchoscopy (n = 118)	Group B: Ultrasound (n = 50)	Group C: Blind (n = 86)	*p*-Value
Any bleeding (minor or major)	27 (22.9%)	11 (22.0%)	20 (23.3%)	0.99
Major hemorrhage requiring transfusion	2 (1.7%)	1 (2.0%)	2 (2.3%)	0.88
Pneumothorax	1 (0.8%)	0 (0%)	3 (3.5%)	0.16
Subcutaneous emphysema	1 (0.8%)	0 (0%)	2 (2.3%)	0.25
Tracheoesophageal fistula	1 (0.8%)	3 (6.0%)	3 (3.5%)	0.29
Cardiac arrest (peri-procedural)	3 (2.5%)	1 (2.0%)	2 (2.3%)	0.97
Total patients with complication	34 (28.8%)	15 (30.0%)	32 (37.2%)	0.42
No complication	84 (71.2%)	35 (70.0%)	54 (62.8%)	—

Note: No statistically significant differences were observed between groups for any complication (χ^2^ or Fisher’s exact tests, *p* > 0.05). Hemorrhage was the most common complication (~22%). Major life-threatening events were rare across all techniques.

**Table 3:** Clinical Outcomes by Technique.

Outcome	Group A: Bronchoscopy (n = 118)	Group B: Ultrasound (n = 50)	Group C: Blind (n = 86)	*p*-Value
Ventilator-free by ICU discharge	73 (61.9%)	32 (64.0%)	50 (58.1%)	0.78
Decannulated (trach removed) in hospital	6 (5.1%)	2 (4.0%)	3 (3.5%)	0.93
ICU Length of Stay (days)	53.5 [17–84]	49.5 [17–166]	32.5 [14–128]	0.017
Hospital Length of Stay (days)	66.0 [23–484]	71.0 [31–212]	58.5 [23–144]	0.046
ICU Mortality	58 (49.2%)	22 (44.0%)	56 (65.1%)	0.025
Hospital Mortality (overall)	89 (75.4%)	34 (68.0%)	75 (87.2%)	0.007
Discharged alive from hospital	29 (24.6%)	16 (32.0%)	11 (12.8%)	0.006
Early mortality (≤72 h after PDT)	5 (4.2%)	2 (4.0%)	8 (9.3%)	0.26

Notes: Ventilator-free = successfully weaned from mechanical ventilation while in ICU (may still have tracheostomy collar). Decannulated = tracheostomy tube removed before discharge (subset of survivors). Length of stay shown for survivors (median [min–max]). Continuous variables were analyzed with ANOVA or Kruskal–Wallis tests, and categorical variables with χ^2^ tests. *p* < 0.05 is considered statistically significant. Early mortality was defined as death occurring within 72 h after PDT.

## Data Availability

De-identified data that support the findings of this study are available from the corresponding author upon reasonable request, subject to institutional approvals and patient privacy regulations.
